# Complete Genome Sequence of *Dyella japonica* Strain A8 Isolated from Malaysian Tropical Soil

**DOI:** 10.1128/genomeA.01164-14

**Published:** 2014-11-13

**Authors:** Raymond K. Hui, Jian-Woon Chen, Kok-Gan Chan, Frederick C. Leung

**Affiliations:** a School of Biological Sciences, the University of Hong Kong, Hong Kong SAR, China; b Division of Genetics and Molecular Biology, Institute of Biological Sciences, Faculty of Science, University of Malaya, Kuala Lumpur, Malaysia; c Bioinformatics Center, Nanjing Agricultural University, Nanjing, China

## Abstract

We previously identified and presented the draft genome of a *Xanthomonadaceae* bacterial strain *Dyella japonica* A8 which shows quorum-quenching activity. Here, we report the complete, closed genome sequence of this bacterium. This complete genome may help to further investigate the comparative quorum-quenching activity among *D. japonica* strains.

## GENOME ANNOUNCEMENT

Intercellular communication among bacterial populations relies on quorum-sensing (QS) mechanisms which involve a number of signaling molecules that initiate coordinated responses across a population (1, 2). The responses triggered via QS signals may directly govern the pathogenesis by synchronizing the production of toxins, proteases, and other immune-evasive factors (3, 4). In addition, such responses may also facilitate the development of biofilm which contributes to the resistance of antimicrobial drugs (5, 6). Recent studies have focused on inhibiting such quorum-sensing pathways, also known as quorum quenching, in order to achieve a therapeutic purpose (7, 8, 9).

A member of the *Xanthomonadaceae* family, *Dyella japonica* strain A8, was isolated from a Malaysian tropical soil layer and was proposed to possess quorum-quenching properties (10). We previously reported the draft genome of this bacterium obtained from shotgun sequencing strategy. In this study, a paired-end library was constructed at 8 kb span with the genomic DNA of *D. japonica* strain A8 previously prepared (10) using the GS FLX Titanium Paired-End Adaptor Set (Roche) according to the manufacturer’s protocol. A shotgun library was also constructed with the GS FLX Titanium Rapid Library Preparation Kit (Roche). The paired-end and shotgun sequencing were performed individually using the 454 GS Junior Platform (Roche) at 200 cycles run module.

Sequences obtained were trimmed and *de novo* assembled using GS De Novo Assembler version 2.8 (Roche). The shotgun and paired-end sequencing runs yielded 158,151 and 140,993 reads, respectively. A total of 24 sequence gaps were identified using Graph theory (11). Primers were designed flanking these gaps and PCR were performed to recover these regions followed by Sanger sequencing using the ABI 3130xl capillary sequencing platform (Life Technologies). *De novo* assembly was performed again including the shotgun, paired-end, and Sanger sequences. A total of 32 contigs were retrieved with an *N*_50_ contig size of 334,110 bp and an average contig size of 178,693 bp, and the largest contig assembled was 933,897 bp. The contigs rendered a coverage estimate of 22.0×, revealing a single-chromosome genome with 4,831,185 bp, comprising a G+C content of 65%. Genome annotation was performed with NCBI Prokaryotic Genome Annotation Pipeline (PGAP) version 2.6 (12). A total of 3,884 genes were identified in the genome, whereas 3,811 predicated coding sequences (CDS) and 16 pseudogenes were revealed. In addition, a total of 13 frameshifted genes were found in the genome. The genome comprises 57 structural RNAs including 50 tRNAs, 6 rRNAs (5S, 16S, 23S), and 1 noncoding RNA (ncRNA). Referring to the Kyoto Encyclopedia of Genes and Genomes (KEGG) database (13), a CDS encoding the *S*-adenosyl-l-homocysteine hydrolase was identified in the genome. This enzyme converts *S*-adenosyl-l-homocysteine (SAH), which is the precursor of quorum-sensing molecule autoinducer-2 (AI-2) and autoinducer-3 (AI-3), into a homocysteine molecule (14). In addition, twelve CDS encoding the LuxR transcriptional regulators and one CDS encoding a carbon storage regulator were also identified in the genome. These findings reveal that the *D. japonica* A8 strain may exert an AI-2-based quorum-sensing mechanism.

### Nucleotide sequence accession numbers.

This whole-genome shotgun sequence has been deposited at DDBJ/EMBL/GenBank under the accession no. CP008884. The version described in this paper is CP008884.1.
